# Maternal Metabolic Health and Mother and Baby Health Outcomes (MAMBO): Protocol of a Prospective Observational Study

**DOI:** 10.2196/72542

**Published:** 2025-04-11

**Authors:** Sarah A L Price, Digsu N Koye, Alice Lewin, Alison Nankervis, Stefan C Kane

**Affiliations:** 1 Department of Medicine University of Melbourne Melbourne Australia; 2 Department of Obstetric Medicine Royal Women's Hospital Melbourne Australia; 3 Department of Diabetes and Endocrinology The Royal Melbourne Hospital Melbourne Australia; 4 Centre for Epidemiology and Biostatistics Melbourne School of Population and Global Health University of Melbourne Melbourne Australia; 5 Department of Obstetrics, Gynaecology and Newborn Health University of Melbourne Melbourne Australia; 6 Maternity Services Royal Women's Hospital Melbourne Australia

**Keywords:** preconception, large for gestational age, small for gestational age, pregnancy outcomes, metabolic disease, diabetes, obesity, hypertension

## Abstract

**Background:**

Metabolic disease is increasingly impacting women of reproductive age. In pregnancy, uncontrolled metabolic disease can result in offspring with major congenital anomalies, preterm birth, and abnormal fetal growth. Pregnancy also accelerates the complications of metabolic diseases in mothers resulting in an increased risk of premature cardiovascular events. Despite the convincing evidence that preconception care can largely mitigate the risks of metabolic disease in pregnancy, there are few data about how to identify the highest-risk women so that they can be connected with appropriate preconception care services.

**Objective:**

The aim of the study is to determine the maternal phenotype that represents the highest risk of having adverse neonatal and maternal pregnancy outcomes.

**Methods:**

This will be a prospective cohort study of 500 women recruited in early pregnancy. The primary outcome is a composite of offspring born small for gestational age (SGA) or large for gestational age (LGA) (customized birthweight ≤10th and ≥90th centile for gestational age). Secondary outcomes are (1) composite of adverse neonatal birth outcomes (SGA, LGA, major congenital abnormalities, preterm birth [<37 weeks’ gestation]) and (2) composite of new maternal metabolic outcomes (gestational diabetes, diabetes in pregnancy, type 2 diabetes [T2D] or prediabetes; gestational hypertension, preeclampsia, eclampsia or new essential hypertension after pregnancy; and gestational weight gain ≥20kg or new overweight/obesity at the 12-18 months postpartum visit). A multivariable logistic regression analysis will be conducted to identify candidate predictors of poor pregnancy outcomes due to metabolic disease. From this model, model coefficients and the associated 95% CIs will be extracted to derive the risk score for predicting the delivery of LGA/SGA offspring (primary outcome) and composites of adverse neonatal outcomes and maternal outcomes (secondary outcomes).

**Results:**

Seed funding for the project was acquired in November 2022 and subsequent funding was acquired in May 2024. The first participant was recruited on March 23, 2023. At the time of manuscript submission, 402 participants have been recruited. Data analysis has not yet been performed. Results are expected to be published in the first half of 2027.

**Conclusions:**

This is a prospective observational cohort study that intends to identify the metabolic disease risk factors, or combination of factors, that are most likely to cause adverse maternal and fetal health outcomes. These characteristics will be used to develop a risk calculator which will assist in identifying the highest risk women and in triaging them to appropriate services. The study has been approved by the institutional Human Research Ethics Committee (HREC/90080/MH-2022).

**Trial Registration:**

Australian New Zealand Clinical Trials Registry ACTRN12623000037606; https://tinyurl.com/yeytsxtp

**International Registered Report Identifier (IRRID):**

DERR1-10.2196/72542

## Introduction

The offspring of mothers with pre-existing metabolic diseases—obesity, diabetes, and hypertension—have an increased risk of significant congenital anomalies and premature birth compared with the offspring of mothers without metabolic disease [1]. These offspring are also twice as likely to develop obesity, diabetes, and hypertension in childhood [2]. For the mothers, pregnancy accelerates the complications of metabolic diseases, resulting in an increased risk of premature cardiovascular events including ischemic heart disease, stroke, and death [3]. Although the consequences of uncontrolled metabolic disease in pregnancy are entirely preventable, our current clinical services are not effective. This is because the highest-risk women are often not identified prior to pregnancy and because high-risk women frequently have poor access to appropriate care.

The women who are most at risk of adverse consequences of metabolic disease in pregnancy are women of low socioeconomic status, women who have experienced trauma, and women living in rural and remote areas [4]. Major barriers for these women accessing and using preconception services include (1) failure to be identified as “high risk” by a health care provider prior to pregnancy; (2) accessing a suitable referral pathway; (3) attending clinic appointments especially if limited by remoteness or finances; and (4) accepting care for metabolic diseases which are often stigmatized diseases and may have implications for the women’s place in her family or wider cultural group. Ironically, there are well-developed services for preconception care of women with severe metabolic disease. However, these services are generally based in tertiary maternity hospitals in metropolitan cities. Due to geographical factors and because referral pathways and clinic entry criteria are often unclear, these services are often used by high socioeconomic status women with a relatively low risk of adverse pregnancy outcomes.

**Figure 1:**
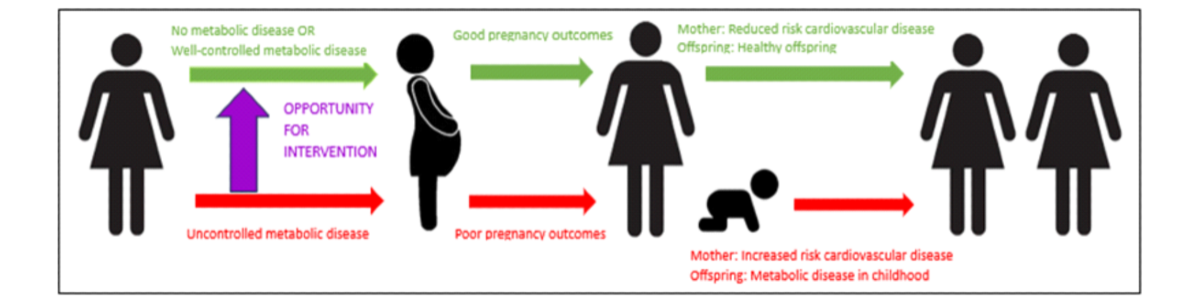
Schematic of the rationale for research study.

We hypothesize that the mothers and offspring at the highest risk of the adverse consequences of metabolic disease are predictable based on the phenotype of the mother prior to pregnancy. Despite the high fetal morbidity that can be associated with metabolic syndrome, little has been done to identify and capture at-risk individuals at a preconception or early antenatal stage. The aim of this study is to develop risk calculators that best predict (1) a mother’s risk of having a neonate with abnormal fetal growth (large for gestational age [LGA] or small for gestational age [SGA]); (2) a mother’s risk of having a serious adverse neonatal outcome; and (3) a mother’s risk of developing new metabolic disease after pregnancy (Figure 1). We will translate this to a user-friendly mobile app that will allow any health care worker to access and triage women to appropriate preconception care.

## Methods

### Study Design and Setting

Study phase I is a multicenter prospective observational cohort study. Study phase II involves the development of risk calculators from the observational cohort established in phase I. The study was prospectively registered with the Australian New Zealand Clinical Trials Registry (ACTRN12623000037606).

**Figure 2:**
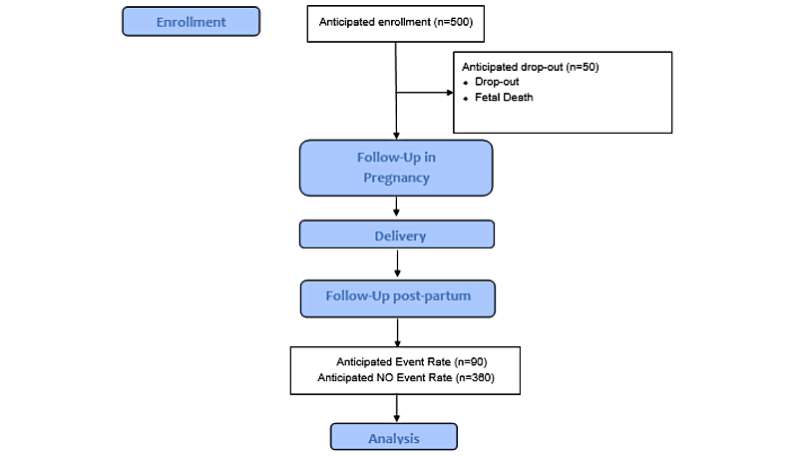
Flowchart of the anticipated study cohort.

The observational cohort study will recruit 500 women who have been referred to either a public or a private tertiary maternity hospital (Figure 2). Both hospitals are tertiary referral centers based in metropolitan Melbourne Australia, and collectively manage >8000 births per year. The recruiting maternity hospitals accept referrals from the entire state of Victoria, such that the hospital populations comprise of metropolitan women and a smaller group of women living in regional and rural areas.

The first participant was recruited on March 23, 2023. The projected timeline for recruitment is that the last participant will be recruited in June 2025. It is anticipated that the last participant will have completed all study visits by June 2026.

### Eligibility and Recruitment

#### Inclusion Criteria

Eligibility for the study will be women aged 18 years and older with pregnancies of 20±4 completed weeks of gestation who provide written consent and are attending a prespecified study site for maternity care. The study will include those with multifetal pregnancies, chronic disease, and those using medications of any type. Women from non–English speaking backgrounds will be included and telephone interpreting services and cultural liaison officers will be used as required.

#### Exclusion Criteria

Women will be excluded from the study if they do not have maternity care at one of the prespecified study sites before 20±4 completed weeks of gestation. Women will also be excluded if they do not wish to provide informed consent or are unable to provide informed consent. This may be due to the inability to read and write, intellectual disability, or a severe active mental illness. It is anticipated that fewer than 1 out of 1000 women screened at maternity hospitals would fulfill this criterion.

#### Withdrawal Criteria

Participants are free to withdraw from the study at any time through revocation of consent (patient preference) or via ceased communication with the recruitment site (lost to follow-up). All data collected prior to the withdrawal of consent will be included in the analysis. Participants who start with maternity care at the recruiting hospitals and then move to seek maternity care elsewhere may continue to take part via telephone/telehealth study visits. Participants who have a pregnancy or neonatal loss will not continue in the study, but information collected until this point will be included in the analysis.

### Recruitment Procedure

Women will be recruited when they attend maternity care visits between 16 and 24 weeks’ gestation. Initial contact will be made by the maternity care provider. Subsequent contact will be made by a dedicated study nurse/doctor at the time of the standard maternity care visit. All participants will receive standard individualized antenatal care based on their clinical needs.

No study activities will be performed before eligibility has been assessed and informed consent has been formally documented. To encourage attendance at clinical visits, parking and meal vouchers will be provided. At the final visit, women will be provided with a comprehensive summary of their metabolic health and a plan for appropriate long-term follow-up.

### Expected Outcomes

#### Primary Outcomes

Metabolically prone offspring defined as a composite of offspring with birthweight ≥90th centile (LGA) and offspring with birthweight ≤10th centile (SGA) based on population-based charts [5,6].

#### Secondary Outcomes

A composite of adverse neonatal outcomes includes the following:

- Offspring with birthweight ≥90th centile on population-based charts (LGA).
- Offspring with birthweight ≤10th centile on population-based charts (SGA).
- Offspring with major congenital abnormalities.
- Offspring born preterm (<37 completed weeks’ gestation).

A composite of new maternal metabolic disease that develops during pregnancy or <18 months postpartum is defined below:

- Gestational diabetes (GDM) as per the International Association of the Diabetes and Pregnancy Study Group (IADPSG)/World Health Organization (WHO) definition (reference); or diabetes in pregnancy as per WHO/IADPSG definition; or previously undiagnosed type 2 diabetes as per American Diabetes Association/WHO definition; or prediabetes as per American Diabetes Association definition (Table S1 in Multimedia Appendix 1).
- Gestational hypertension; or pre-eclampsia; or eclampsia; or new essential hypertension after pregnancy (Table S2 in Multimedia Appendix 1).
- Gestational weight gain ≥20 kg or new overweight/obesity after pregnancy (BMI ≥25 kg/m^2^ and body weight increase of ≥5 kg compared with prepregnancy weight) at the 12-18 months postpartum visit.

### Clinical Assessments

A schedule of assessments and measurements is presented in Table 1.

**Table 1:** Summary of study activities.

	Visit 1 (20, SD 4 weeks gestation)	Visit 2 (36, SD 4 weeks gestation)	Visit 3 (Peripartum)	Visit 4 (12-18 months postpartum)
Informed consent and eligibility	✓	✓		
Collect basic demographic details including postcode	✓			
Collect past medical, surgical, obstetric, and gynecological	✓			
Collect new medical, surgical, obstetric, and gynecological		✓	✓	✓
Adverse events		✓	✓	✓
Medication	✓	✓	✓	✓
Allergies	✓	✓	✓	✓
Smoking, drug, and alcohol use history	✓			
Maternal Anthropometry				
Height	✓			
Weight	✓	✓		✓
Waist circumference	✓	✓		✓
Bioimpedance	✓	✓		✓
Blood tests				
Glucose, insulin, and c-peptide	✓	✓		✓
HbA_1c_^a^	✓	✓		✓
CRP^b^	✓	✓		✓
Leptin	✓	✓		✓
Provide maternal and neonatal mouth swabs to participants		✓		
Collect 75g OGTT^c^ result		✓		✓
Collect obstetric ultrasound results		✓		
Collect maternal and neonatal mouth swabs for epigenetic analysis		✓	✓	
Collect obstetric medical records including the birth summary		✓	✓	
Collect height and weight data from the childhood development book (“Baby book”)		✓		✓

^a^HbA_1c_: hemoglobin A_1c_.

^b^CRP: C-reactive protein.

^c^OGTT: oral glucose tolerance test.

#### Medical History and Medications

At the first visit, a full medical history will be taken by a trained clinician including past medical history and surgical history. Outcomes of previous pregnancies will be recorded including outcome of the pregnancy, gestation, pregnancy complications, and (if relevant) birthweight and neonatal complications. Current medications including dose, and dosing schedule will be recorded. Smoking, alcohol, and recreational drug use will be recorded.

#### Blood Pressure

Blood pressure will be measured after 5 minutes of rest in a seated position using an appropriately sized cuff on the bared left upper arm supported at heart level. Auscultatory readings with an aneroid sphygmomanometer and stethoscope or automated oscillometric readings with a single read per activation device will be collected by trained operators**.**

#### Weight, Height, and Waist Circumference

Weight will be measured by a trained technician on calibrated digital scales with an accuracy of ±0.01 kg. Participants will be asked to wear light clothing but no shoes. Height will be measured by a trained technician using a standing stadiometer with an accuracy of ±0.5 cm. Waist circumference will be measured with an accuracy of ±0.5 cm using a standard tape measure according to the WHO guidelines (at the end of a normal expiration, at the midpoint between the lower margin of the last palpable rib and the top of the iliac crest, as the average of 2 measurements). BMI will be calculated by dividing weight in kg by height in meters squared.

#### Bioimpedance Analysis

Bioimpedance will be performed using the DC-430MAS Tanita Dual Frequency Body Composition Analyser. Data obtained from bioimpedance analysis will include fat mass (%), fat mass (kg), free fat mass (kg), muscle mass (kg), and bone mass (kg).

#### Laboratory Assays

Phlebotomy is a low-risk procedure. At each visit, the required blood samples will be taken for glucose, insulin, C-peptide, high-sensitivity C-reactive protein, and leptin using an aseptic technique. Samples will be mixed by inversion and centrifuged at 4 °C. Plasma from the tubes will be aliquoted into 5 microtubes with 2 spare microtube samples. Plasma samples will be batched for analysis to minimize interassay error. The sample will be stored at –80 °C before analysis. Spare tubes will only be used in the event of a processing error.

Samples will be tested by commercial laboratories accredited by the National Association of Testing Authorities (Australia) and The Royal College of Pathologists of Australia for compliance with National Pathology Accreditation Advisory Council (Australia) standards and ISO (International Organization for Standardization) 15189. Test results were interpreted in accordance with normative reference values. The study database allows the management of the samples.

#### Calculated Insulin Resistance

Homeostasis model assessment of insulin resistance (HOMA-IR) values will be calculated by the equation (fasting insulin [μU/mL] × fasting glucose [mM])/22.5. Insulin resistance is defined by a HOMA value >4 (reference).

#### Oral Glucose Tolerance Test Results and Other Forms of Testing for Gestational Diabetes

The 75-g oral glucose tolerance test (OGTT) is a standard of care to screen for GDM at 24-28 weeks’ gestation in Australia. A 75-g OGTT may also be performed at 10-20 weeks of gestation if there are risk factors for GDM. A diagnosis of GDM may also be made using other methods such as HbA_1c_ (hemoglobin A_1c_), glucose readings from a period of fingerstick monitoring, or by evaluating continuous glucose monitoring CGM) metrics. If the woman is considered to have GDM by the treating clinical team, she will also be given this diagnosis for the purpose of the study. All medications used to treat GDM will be recorded.

For all such women, a 75-g OGTT or some other form of testing for diabetes should be repeated >6 weeks’ post partum and these results will also be collected.

#### Obstetric Ultrasound

Obstetric ultrasounds are routinely performed in Australia to assess fetal morphology at 20-22 weeks’ gestation. The fetal morphology scan report will be collected from all participants. Fetal growth scans may be performed at 28, 32, or 36 weeks for obstetric indications. If growth scans are performed, the estimated fetal weight and abdominal circumference will be collected from ultrasounds that have been formally reported. The reported customized growth centiles are based on GROW customized charts which are customized for known constitutional variables (maternal height, weight, ethnic origin, and parity) but not pathological factors such as smoking and diabetes. This method of customization has been demonstrated to classify the greatest number of offspring at risk of perinatal mortality [7-9]. Any congenital anomalies reported on ultrasound will also be recorded.

#### Obstetric Discharge Summary

All neonates born at recruiting maternity hospitals have a standard obstetric discharge summary on discharge from the hospital. Neonatal information collected will include date of birth, gestation at delivery, birthweight, birthweight centile, sex, Apgar’s score, neonatal complications (hypoglycemia, jaundice, congenital anomalies [major/minor], and others). Maternal information will include mode of delivery, and maternal pregnancy complications (GDM, gestational hypertension/preeclampsia, and others).

#### Cheek Swab (for Epigenetic Analysis)

Swabs will be taken from mother and baby using a sterile regular flocked dry swab using a kit supplied at 36 (SD 4) weeks of gestation. These samples will be self-collected by rubbing the swab against the inside of the cheek 10 times and reinserting the swab into the plastic applicator. Samples will be given to nursing staff before discharge. The swabs will be sent to pathology for long-term storage until all samples are collected.

### Sample Size

We estimate recruitment of 500 participants over 24 months, with an estimated dropout of 5% inclusive of dropouts due to fetal/neonatal death. The primary outcome variable of interest is LGA/SGA offspring (yes/no) due to the known association with short- and long-term metabolic consequences and the fact that most babies with congenital anomalies and prematurity will have abnormal fetal growth. By definition, LGA (<90th centile for gestational age) and small for gestational age (>10th centile for gestational age) occurs in 20% of the population. With 475 participants (500 minus 25 dropouts), we would expect 95 offspring of interest compared with 380 normal-weight offspring.

Sample size calculation for the multivariable prediction modeling was conducted using the pmsampsize package in Stata (version 17.0; StataCorp) according to the 3 criteria (shrinkage, overfitting, and precision). From prior data, it is anticipated that the primary outcome of interest (presence of LGA/SGA offspring) will occur in 20% of the population. Åmark et al [10] reported an area under the receiver operating characteristics curve (AUC) of 0.8 from a logistic regression-based prediction model. Therefore, a minimum sample size of 440 is required with at least 88 events to ensure the expected shrinkage required is 10% or less (to minimize the potential overfitting), and a small absolute difference of 0.05 in the model’s apparent and adjusted Nagelkerke *R*^2^ value.

We anticipate that ≈20% of women will develop a maternal metabolic disease of interest including ≈15% who develop diabetes, ≈5% who gain excessive weight (>20 kg), and ≈15% who develop a hypertensive disease of pregnancy. Given the anticipated overlap of these conditions, ≈20% (n=90) of women will have at least one maternal metabolic disease of interest (secondary outcome).

### Methodology

#### Data Collection

Study data are collected using paper case report forms. These paper documents will be kept in a locked cupboard accessible only to local research staff. Patient information is collected and stored by the investigators in a confidential REDCap (Research Electronic Data Capture system; Vanderbilt University), with password protection and restricted access. This will include patient information transcribed from the hospital’s electronic medical record. REDCap is a secure, web-based app designed for research studies, providing a validated data entry interface, audit trails for data tracking, automated export procedures, and secure procedures for data import [11]. Internet access to the REDCap database will use a secure server located at the University of Melbourne, Australia (REDCap consortium host). Access will be limited to local research staff, approved data administrators, and project statisticians.

All biological samples will be labeled with a reidentifiable study number. The electronic study information will be stored in a dedicated, limited-access clinical database. Only study investigators and key study staff will have access to the database. Biological samples will be stored at –80 °C until all samples are collected. They will then be processed in batches by the local pathology network according to standard protocols.

Epigenetic samples (buccal swabs) will be stored at –80 °C until all samples are collected. DNA will then be extracted using standard processes for DNA extraction, and samples will be prepared for shipping. Methylation analysis will occur at an external genomics facility [12,13]. Once processed, data will be interpreted with the assistance of appropriate clinicians at Murdoch Research Institute. These samples are hypothesis-generating only and will not be included in the prediction modeling.

#### Data Management

Management of comprehensive and valid records will be the responsibility of each site. To ensure a systematic approach to data collection, the same team of study nurses will work between the sites using the same protocol and standard operating procedures. Data integrity will be maintained through a review of the collected data prior to, during, and after each study visit. Medication and adverse event data will be reviewed during each study visit. In addition, every 3 months the study coordinator will review the data collected to ensure completeness and consistency of data. Any data discrepancies will be queried with the participant by email or telephone within 4 weeks of being noted. All data, including the attendance of study visits, will be captured on a REDCap database. This will ensure that the study can meet regular deadlines required for process reporting as required by the Human Research Ethics Committee.

#### Data and Safety Monitoring

We anticipate very few study-related adverse events or serious adverse events. The only anticipated adverse events are phlebotomy-related side effects (ie, bruising, bleeding, vasovagal syncope) and a negative psychological response to clinical visits (ie, having weight measured). These events will be circumvented by having provided participants with a full and comprehensive review of study visits at the time of enrolment and prior to each study visit.

Reports of measurements taken at the time of clinical visits including weight and bioimpedance data, and the results of laboratory tests that are clinically relevant, will be made available to participants. Results that are not clinically relevant will not be disclosed to participants as they will not impact clinical care and may be difficult to interpret (ie, lack of reference ranges in pregnant women). Any concerning results will immediately be relayed to the treating maternity team. All other results will be reviewed within 1 week. The outcomes of participants will be discussed with the chief investigator each month. Any systematic problems with data management and adverse event monitoring or reporting will be rectified. REDCap queries will be addressed monthly by the study coordinator and reviewed quarterly by the study statistician.

### Ethical Considerations

This is a prospective observational cohort study that intends to identify the metabolic disease risk factors, or combination of factors, that are most likely to cause adverse maternal and fetal health outcomes. No study-related adverse events are anticipated. The study has been granted ethics approval from the Royal Melbourne Hospital Human Research and Ethics Committee (HREC/90080/MH-2022) and recruitment is ongoing. Each participant will provide written informed consent before any study activities are undertaken and participants are free to withdraw consent at any time. Confidentiality will be maintained and only group data will be presented. Dissemination will be through peer-reviewed publications at national and international conferences, national and international obstetric medicine societies, and specialist colleges.

### Statistical Methods

This study will follow the Transparent Reporting of a Multivariable Prediction Model for Individual Prognosis or Diagnosis reporting guidelines for prognostic studies [14]. Summary characteristics of the study cohort will be described using means (with SD) or medians (with IQRs) for continuous variables, depending on the shape of the distribution, and numbers and percentages for categorical variables.

A risk calculator will be developed to determine the risk of an abnormally grown fetus (ie, the primary outcome of the study). Participant data will be split randomly into either training (70%) or validation (30%) datasets according to standard prediction model methods. Multivariable logistic regression analysis will be conducted to identify candidate predictors of poor pregnancy outcomes due to metabolic disease. After starting with the most comprehensive model that includes all potential risk factors for an LGA or SGA offspring (ie, the primary outcome). These include categorical variables including maternal SES (based on postcode), maternal parity, maternal smoking status, maternal obesity class, maternal preexisting diabetes, maternal preexisting renal disease, essential hypertension, use of antihypertensive medication, use of metformin, gestational hypertension, preeclampsia/eclampsia, previous LGA/SGA offspring, major congenital anomaly, and continuous variables including maternal age, maternal gestational weight gain, maternal glycemic control (HbA_1c_), total daily dose insulin, maternal systolic blood pressure, maternal diastolic blood pressure, birthweight of previous offspring, fasting glucose, plasma insulin, plasma C-peptide, plasma leptin, HOMA-IR.

A backward selection method will be performed to determine which combination of risk factors generates the most parsimonious predictive model. The number of candidate variables will be limited to data collected in the dataset. From this parsimonious model, model coefficients and the associated 95% CIs will be extracted to derive the risk score for predicting the delivery of LGA/SGA offspring. Internal validation of the model will be performed using 5-fold cross-validation and bootstrap validation. The performance of the model will be assessed using the AUC and calibration slope.

A similar approach will be taken for the secondary outcomes, and these models will be used to develop risk calculators for a composite of neonatal adverse outcomes and a composite of maternal adverse outcomes. Ongoing statistical input will be provided by the University of Melbourne.

## Results

This project was funded by a National Health and Medical Research Council Investigator Grant awarded in November 2021 (CIA Price 2022-2026; 2007957), and by a Translational Challenge Grant from the Ramsay Hospital Research Foundation awarded in April 2024 (CIA Price 2024-2027; 2023/TCG/0038). The first participant was recruited on March 23, 2023. At the time of manuscript submission, 402 participants have been recruited. The projected timeline for recruitment is that the last participant will be recruited in June 2025. Therefore, it is anticipated that the last participant will have completed all study visits by June 2026. Data analysis has not yet been performed. Results are expected to be published in the first half of 2027.

## Discussion

### Why Predict Adverse Pregnancy Outcomes?

Uncontrolled maternal metabolic disease can profoundly impact both maternal and neonatal outcomes. Major congenital anomalies due to maternal metabolic disease are a common cause of neonatal death and long-term disability. For example, the risk of a major congenital anomaly such as congenital heart defect or spina bifida is 25% with suboptimally controlled diabetes (HbA_1c_ >10%) compared with 3% with well-controlled diabetes (HbA_1c_ <7%) [15,16]. Similarly, preterm birth can result in long-term neurodevelopmental issues and poor academic outcomes [17].

Even in children born at term, abnormal fetal growth can result in a long-term predisposition to metabolic disease. Around half of children born LGA (>90th centile for gestational age) or SGA (<10th centile for gestational age) [16] go on to develop metabolic syndrome (obesity, dysglycemia, and hypertension) by school age [2] with subsequent deterioration of metabolic disease in early adolescence [18]. This occurs due to a combination of factors including altered body composition at birth, altered appetite at birth, and atypical organ development including a lower endowment of cardiac myocytes, pancreatic beta cells, and nephrons [19].

For women, metabolic disease during pregnancy is an independent risk factor for later cardiovascular disease [20]. In the decade after preeclampsia, a meta-analysis including 200,000 women demonstrated a 4-fold increase in the relative risk of developing chronic hypertension and a 2-fold increase in the relative risk of ischemic heart disease and stroke [21]. For women who develop GDM, the lifetime risk of T2D is increased by 7-fold [22]. Lee et al [23] found that all-cause mortality was increased in women who were obese during pregnancy (BMI>30 kg/m^2^) versus normal BMI after adjustment for confounding factors (hazard Ratio 1.35, 95% CI 1.02-1.77). Pregnancy loss due to late first-trimester miscarriage or stillbirth is also associated with increased cardiovascular disease in the mother, likely because pregnancy unmasks an underlying endothelial dysfunction [15].

### Why Existing Prediction Models Are Inadequate

There are very few studies that have aimed to predict adverse pregnancy outcomes based on the maternal phenotype, and most of these have used maternal characteristics in combination with obstetric tests such as biomarkers in blood or ultrasound [24-27]. To the investigator’s knowledge, there has only been one study that has used maternal prepregnancy characteristics to predict the risk of poor pregnancy outcomes [28].

The Generation R study group used easily obtainable maternal preconception characteristics including age, ethnicity, parity, BMI, and smoking status to model the risk of offspring born LGA/SGA. Basic models demonstrated an AUC of 0.63 (95% CI 0.61 to 0.65) and 0.64 (95% CI 0.62 to 0.66) for preterm birth/SGA and LGA, respectively. Interestingly, more complex models involving sociodemographic and dietary details only led to small improvements in the model [28].

However, the Generation R study was a population-based prospective cohort study that aimed to identify early and genetic causes and casual pathways leading to abnormal growth and development of the offspring [29]. Risk prediction modeling in this study focused on all potential causes of LGA and SGA in a whole population cohort. This study did not specifically focus on LGA/SGA in the context of metabolic disease, nor did it consider the metabolic outcomes of the mother. Women with metabolic disease are known to be at higher risk of adverse pregnancy outcomes than the general population [30]. This cohort requires information about the risk of poor pregnancy outcomes both for themselves and their offspring.

Within an established pregnancy, Frick et al [31] found that LGA offspring (>95th centile) could be predicted based on increasing maternal BMI, diabetes status, and the presence of chronic hypertension. However, this prediction was less accurate in non-Caucasian women, in smokers, and in nulliparous women. In parous women, LGA offspring were predicted based on the birth-weight Z-score of previous offspring, a prior history of GDM, and a decrease with interpregnancy interval. This study then refined the prediction model using early fetal ultrasonography [31].

A population-based study from Ontario Canada (n=634,290) found that it was possible to predict and prevent severe maternal morbidity with moderate discrimination. Those women who had poor short-term pregnancy outcomes were older, had more medical comorbidities, and conceived using assisted reproductive technologies [32]. Similarly, Akinci et al [33] reported that in a small cohort (n=164), prepregnancy obesity, greater gestational weight gain, and a fasting glucose level (>5.5 mmol/L) on the 75-g OGTT predicted the development of metabolic syndrome in postpartum women with moderate to high discrimination.

### What the Metabolic Health and Mother and Baby Health Outcomes Study Will Contribute

There are very few studies that use maternal characteristics to predict both maternal and fetal/neonatal pregnancy outcomes, even though they are inextricably linked. There are also few studies that consider the impact of metabolic disease as a whole rather than focusing on individual components of metabolic disease- hypertension, obesity, or dysglycemia.

We anticipate the Metabolic Health and Mother and Baby Health Outcomes (MAMBO) project will be able to fill this gap by developing a risk prediction tool that (1) considers how multiple maternal metabolic risk factors interact to result in adverse pregnancy outcomes, and (2) considers both maternal and neonatal pregnancy outcomes.

A risk calculator that can be used as a mobile app would provide health care workers with a simple effective tool to objectively calculate pregnancy risk and to triage women to appropriate services based on a standard dataset. Health care providers would require very little background knowledge to use the risk calculator. However, the tool would produce personalized data about the risks of an adverse pregnancy outcome. This would assist health care workers in effectively communication with the woman, her family, and with other health care providers.

### Limitations

Ideally, the MAMBO cohort would have been recruited prior to conception. However, this would have required a very large sample size to allow for women who did not conceive in a specified timeframe and for additional dropouts due to the lag time between recruitment and conception. Therefore, the pragmatic decision was made to recruit women at 20±4 weeks’ gestation. Existing data demonstrates a strong correlation between maternal anthropometric data and biomarkers in early pregnancy and the prepregnancy period [34].

The MAMBO cohort consists entirely of women attending major metropolitan maternity hospitals. Although these hospitals accept referrals from primary care and smaller hospitals that provide maternity care for women living in rural and remote locations, nonmetropolitan women are likely to be underrepresented in this cohort. Women from minority culture groups and women from non–English speaking backgrounds may also be less likely to participate in a prospective cohort study. To address these issues, translators and cultural liaison officers will be made available. We will aim to pair study visits with usual maternity care appointments. The final clinical study visit can be performed by phone if necessary.

### Outcomes and Significance

Growing numbers of women are impacted by metabolic disease when they pursue pregnancy. However, women of low socioeconomic status, non-English speaking backgrounds, and women living in rural and remote areas are less likely to access preconception care services. Therefore, these women are at a greater risk of short-term adverse pregnancy outcomes, and long-term due to metabolic programming in the infant and unmasking of endothelial dysfunction in mothers.

The development of a risk calculator that predicts the women most likely to have poor pregnancy outcomes may assist in preventing these adverse pregnancy outcomes. It would allow women to be triaged by any health care professional and would ensure that the highest risk women are identified and have the opportunity to be referred to high-quality preconception care services and to receive information on how to prevent adverse health outcomes for themselves and their offspring. The risk calculator also has the potential to act as a platform that assists health care professionals in providing lower-risk women with basic preconception care information.

### Conclusions

Currently, the women at the highest risk of adverse pregnancy outcomes due to metabolic disease are the least likely to access preconception health interventions. The MAMBO project intends to bridge this gap by developing a risk prediction tool that allows any health care practitioner to accurately assess risk, and to triage women to appropriate preconception care services. In this way, we aim to prevent adverse maternal and fetal health outcomes.

## Appendix

Multimedia Appendix 1 Additional tables.

## Abbreviations

AUC: area under the receiver operating characteristics curve
GDM: gestational diabetes
HbA_1c_: hemoglobin A_1c_
HOMA-IR: homeostasis model assessment of insulin resistance
IADPSG: International Association of the Diabetes and Pregnancy Study Group
LGA: large for gestational age
MAMBO: Metabolic Health and Mother and Baby Health Outcomes
OGTT: oral glucose tolerance test
REDCap: Research Electronic Data Capture
SGA: small for gestational age
T2D: type 2 diabetes
WHO: World Health Organization

## Data Availability

The datasets generated during this study are available from the corresponding author on reasonable request.
