# Supplementary material for: Maternal Metabolic Health and Mother and Baby Health Outcomes (MAMBO): Protocol of a Prospective Observational Study
Source: JMIR Res Protoc. 2025 Apr 11;14:e72542. doi: 10.2196/72542 (PMC12032496; doi:10.2196/72542)
Supplement: Multimedia Appendix 1 [file resprot_v14i1e72542_app1.docx]

**Table S1**: Definition of Diabetes and Pre-Diabetes for Patient Outcomes

|  | **Defining Body** | **Timing of Test** | **Criteria** |
| --- | --- | --- | --- |
| Gestational Diabetes (GDM) | IADPSG/WHO | 10-28 weeks’ gestation | 75g OGTT;  0-hour plasma glucose: ≥5.1 mmol/L  1-hour plasma glucose: ≥10.0 mmol/L  2-hour plasma glucose: ≥8.5 mmol/L |
| Diabetes In Pregnancy (DIP) | IADPSG/WHO | 10-28 weeks’ gestation | 75g OGTT:  0-hr plasma glucose: ≥7.0 mmol/L  2-hour plasma glucose: ≥11.1 mmol/L  OR  HbA1c ≥48 mmol/mol (6.5%) |
| Type 1 Diabetes (T1D) | WHO | Any time | Autoantibodies and/or low C-peptide  AND  75g OGTT:  0-hr plasma glucose: ≥7.0 mmol/L  2-hour plasma glucose: ≥11.1 mmol/L  OR  HbA1c ≥48 mmol/mol (6.5%) |
| Type 2 Diabetes (T2D) | WHO/ADA | >6 weeks post-partum | 75g OGTT:  0-hr plasma glucose: ≥7.0 mmol/L  2-hour plasma glucose: ≥11.1 mmol/L  OR  HbA1c ≥48 mmol/mol (6.5%) |
| Pre-Diabetes | WHO/ADA | >6 weeks post-partum | 75g OGTT:  0-hr plasma glucose: 5.6-6.9 mmol/L  2-hour plasma glucose: 7.8-11.0 mmol/L  OR  HbA1c 39-46 mmol/mol (5.7-6.4%) |

*Nb. OGTT oral glucose tolerance test; IADPSG International Diabetes In Pregnancy Study Group; WHO World Health Organisation; ADA American Diabetes Association.*

**Table S2:** Definitions of Pregnancy-Associated Hypertension for Patient Outcomes

|  | **Defining Body** | **Timing of Test** | **Criteria** |
| --- | --- | --- | --- |
| Gestational Hypertension | SOMANZ [1] | >20 weeks gestation- 3 months post-partum | New onset of hypertension (systolic blood pressure ≥140 mmHg and/ or diastolic blood pressure ≥90 mmHg) after 20 weeks gestation without any maternal or fetal features of preeclampsia, followed by the return of blood pressure to normal within 3 months post-partum. |
| Preeclampsia | SOMANZ [1] | >20 weeks gestation- 3 months post-partum | New onset of hypertension (systolic blood pressure ≥140 mmHg and/ or diastolic blood pressure ≥90 mmHg) >20 weeks gestation accompanied by one or more signs of new onset organ involvement:  (i)Renal: PCR ≥ 30mg/mmol; Serum Cr > 90 μmol/L  (ii)Liver involvement: Raised transaminases  (iii)Haematological: Platelets <150,000µ/l, haemolysis (low haptoglobin with or without fragmentation, elevated LDH), disseminated intravascular coagulation  (iv)Neurological: Convulsions, cerebral instability, cerebrovascular accident  (v)Pulmonary oedema  (vi)Features of placental dysfunction: Sonographic features of fetal growth restriction, sFlt-1/PlGF ratio has not excluded preeclampsia |
| Essential Hypertension | SOMANZ [1] | Pre-pregnancy, <20 weeks gestation, or >3 months post-partum | Hypertension (blood pressure greater than or equal to 140 mmHg systolic and/or 90mmHg diastolic). |

*Nb. SOMANZ Society of Obstetric Medicine*

**References**

1. Shanmugalingam R, Makris A. A summary of the 2023 Society of Obstetric Medicine of Australia and New Zealand (SOMANZ) hypertension in pregnancy guideline. Med J Aust. 2024 Dec 27. PMID: 39726406. doi: 10.5694/mja2.52576.
